# Advancing equity in cross-cultural psychology: embracing diverse epistemologies and fostering collaborative practices

**DOI:** 10.3389/fpsyg.2024.1368663

**Published:** 2024-04-04

**Authors:** Gulnaz Anjum, Mudassar Aziz

**Affiliations:** Department of Psychology, University of Oslo, Oslo, Norway

**Keywords:** inclusivity in cross-cultural psychology, knowledge co-production, equitable collaborations, local epistemologies, representation in research, publication disparities

## Abstract

Psychology, and cross-cultural psychology (CCP) in particular, plays a pivotal role in understanding the intricate relationship between culture and human behavior. This paper sheds light on the challenges of inequity and marginalization, especially concerning scholarship from the Global South, which have roots in historical colonial practices. It highlights how intellectual extractivism and the predominance of Western research methodologies often overlook the contributions of Global South scholars and indigenous ways of knowing. Such imbalances risk narrowing the scope of psychological inquiry, privileging American and European perspectives, and undermining the richness of global human experiences. This paper calls for a shift toward more equitable collaborations and the recognition of diverse epistemologies. By advocating for genuine representation in research and valuing local knowledge, it proposes pathways for a more inclusive and authentic exploration of human behavior across cultures.

## Introduction

Psychology as a discipline, and Cross-cultural psychology (CCP), as a subdiscipline seek to discern universal patterns of behavior and understand the variations of these patterns across different cultural settings. Rooted in the notion that culture significantly impacts psychological processes, cross-cultural psychology fundamentally acknowledges that human behaviors, thoughts, and emotions are intricately intertwined with the societal and cultural context in which individuals are embedded (Berry et al., 2011). It does not merely juxtapose behaviors from different cultures against one another; instead, it seeks to understand underlying cultural reasons, values, beliefs, and practices that may explain the observed variations.

Research in CCP has been historically dominated by Western perspectives, methodologies, and interests, often at the expense of non-Western cultures and knowledge systems (Adams and Markus, 2004; Henrich et al., 2010a,b). A comprehensive review is imperative to critically assess and address this imbalance, ensuring that the field evolves to be more representative and inclusive of diverse cultural contexts. Ethical considerations in psychological research are paramount. The historical intellectual extractivism is still sometimes present in research practices, particularly in relation to communities in the Global South, raising significant ethical concerns (Teo, 2010). This review would provide a platform to interrogate these practices and advocate for more ethical, respectful, and reciprocal research methodologies. Indigenous knowledge systems and local epistemologies are often marginalized in mainstream CCP research (Smith, 2012), it would advocate for their inclusion and recognition in the broader psychological discourse.

Decolonizing psychological science is a multifaceted endeavor that requires a comprehensive and critical examination of the epistemologies, and practices that have traditionally shaped the field. The literature from Adams, Arnett, Barrero, and others offers a rich tapestry of insights and recommendations for this transformation. This review synthesizes these insights and suggests pathways for a more inclusive and globally representative psychological science.

The acronym WEIRD – standing for Western, Educated, Industrialized, Rich, and Democratic – describes populations that, despite being the most frequently studied in psychological research, do not represent the global majority. This concept highlights a pervasive bias within psychological studies, emphasizing the need to scrutinize the generalizability of research findings derived predominantly from WEIRD populations to those outside this narrow scope (Henrich et al., 2010a,b). In contrast, the term “Majority World” encompasses countries and populations that, while forming the bulk of the world’s demographic, remain largely marginalized or underrepresented within global economic structures and scholarly discourse. This notion challenges traditional “developed” versus “developing” country dichotomies by foregrounding the demographic weight and advocating for enhanced representation and voice of these populations in global dialogs, including research and policy formulation. It aims to recalibrate perceptions of global inequality, shedding light on the shared experiences of a significant portion of humanity (often coinciding with the Global South) in a way that underscores their agency and multifaceted identities (Alam, 2007; Khan et al., 2022).

Adams and Markus (2004) and Adams et al. (2015) emphasize the importance of a cultural psychology approach and decolonizing research methods. They argue for the necessity of acknowledging and integrating indigenous knowledge systems and methodologies in psychological research. This entails moving beyond mere inclusion to a profound respect and partnership with indigenous knowledge holders, ensuring that research is not only about them but with and by them. Arnett’s (2002, 2008) work on the psychology of globalization and the call for American psychology to become less American underscores the urgency of expanding the cultural and geographic scope of psychological research to include the “neglected 95%” of the world’s population.

Arnett (2008) and Thalmayer et al. (2021) reveal enduring underrepresentation in psychological research, with over 95% of samples across 2003–2018 drawn from Western, Educated, Industrialized, Rich and Developed (WEIRD) societies, predominantly the United States. Their analyses highlight minimal increases in diversity, from 3 to 4%, in studies involving non-WEIRD populations, mainly from Confucian East Asian backgrounds, while research including African, Latin American, Middle Eastern, and other cultures remains notably rare. This consistency underscores the field’s slow progress toward inclusivity and geographical diversity in psychological research (Krys et al., 2024).

### The dichotomy of the global north and global south

The terms “Global North” and “Global South” are used to describe a geopolitical and economic division between countries, transcending mere geographical distinctions. The “Global North” typically includes countries that are wealthier, more industrialized, and often located in the Northern Hemisphere, but not exclusively so. These countries have historically had a larger influence on global economic policies and knowledge production. Conversely, the “Global South” refers to nations that are generally less economically developed, often (but not exclusively) situated in the Southern hemisphere and have historically been marginalized within global economic and political systems (Connell, 2007; Omotayo Oladejo et al., 2024).

The dichotomy of the Global North and Global South, while serving as a heuristic tool in our discussion, requires a nuanced understanding that acknowledges its limitations. This framework is employed not to oversimplify the rich diversity within these broadly defined regions but to highlight the systemic inequalities and historical legacies that influence psychological research and knowledge production. The Global North–South distinction facilitates a critical examination of how colonial legacies and economic power dynamics shape the epistemological diversity within psychology (Connell, 2007; Teo, 2018; Barnwell and Wood, 2022; Omotayo Oladejo et al., 2024).

It is imperative to underscore that this categorization is not a mere geographic distinction but rather a reflection of the complex interplay of developmental trajectories, colonial histories, and socio-economic contexts that define each region’s unique place in the global hierarchy. For instance, countries such as Australia and New Zealand, despite their geographical positioning in the Southern Hemisphere, are typically aligned with the Global North due to their higher levels of industrialization, economic stability, and historical roles as colonizers rather than the colonized. Conversely, nations like Bangladesh and Myanmar, situated in the geographical Northern Hemisphere, are categorized within the Global South framework. This classification acknowledges their experiences with colonial subjugation, ongoing developmental challenges, and the struggle for equitable participation in global knowledge production and economic systems. This nuanced understanding is vital, as it highlights that the Global North and South dichotomy transcends mere physical geography, delving into the realms of socio-economic inequalities, historical legacies, and the quest for a more equitable global order.

The use of “Global North” primarily refers to regions and countries historically characterized by colonialism and imperialism, and which currently exhibit significant economic and educational advantages in the global context. Conversely, “Global South” encompasses countries that have historically been colonized or are currently experiencing lower levels of industrialization and economic stability, thereby facing systemic disadvantages in global knowledge production (Quijano, 2000; Bhambra, 2014). By critically engaging with this dichotomy, we aim to underscore the importance of addressing these global imbalances and inequities to foster a more equitable and inclusive psychological science. This entails promoting collaborative and reciprocal research partnerships that respect the sovereignty and epistemological contributions of scholars and communities from the Global South (Mignolo, 2012; Smith, 2021).

In this paper, the adoption of “Global North and South” terminology over “East versus West” or “WEIRD versus non-WEIRD” in psychological discourse is done after a comprehensive reflection. This reflection by the scholars from the Global South, scholars of color, both from former colonies of Britain was done based on their lived experience of intergenerational. Historical, cultural, economic, and perceived power dynamics disparities inherent in global academic research. This dichotomy, rooted in colonial legacies, transcends geographical distinctions to encapsulate economic, political, and social disparities that have historically shaped and continue to influence resource distribution, power structures, and knowledge production systems (Quijano, 2000; Mignolo, 2012). It recognizes the “coloniality of power” and its persistent impact on contemporary societies, especially in psychological science, where Global North’s predominant theories and methodologies may not universally apply or accurately represent Global South populations.

Furthermore, the Global North–South categorization captures a broader spectrum of socio-economic and cultural conditions compared to the East–West divide, which often overlooks internal diversities within these regions, or the WEIRD framework, which, while highlighting the overrepresentation of Western populations in research, falls short of addressing deeper economic and geopolitical disparities (Henrich et al., 2010a,b). The North–South perspective thus offers a nuanced framework for discussing asymmetries in knowledge production and advocating for a more inclusive, equitable representation in psychological research. It urges the field toward embracing diverse epistemologies and methodologies, fostering a global psychology that values contributions from across the economic spectrum, particularly those emanating from the underrepresented Global South. This approach not only aims to rectify historical and ongoing imbalances but also enriches psychological science with a multiplicity of perspectives and insights.

The historical context of colonial expropriation and its role in shaping the economic activities associated with the Industrial Revolution illustrates the foundational disparities between the Global North and South. The reliance on colonial extractivism, exemplified by the cotton gin’s dependence on unpaid slave labor, underscores the economic dynamics that facilitated wealth accumulation in the Global North at the expense of the Global South (Amin, 2014; Moore, 2015).

Contemporary economic repercussions of these historical processes are evident in the disproportionate impacts of climate change and resource depletion on the Global South (Arrow et al., 2004; Gaffney, 2014). The acknowledgment of corporations based in the Global North contributing significantly to global greenhouse gas emissions highlights the continued exploitation and economic degradation facilitated by colonial capitalism (Maitland et al., 2022). While we use this categorization, it has similar challenges to other categorizations (East/West; North/South), we assert that a more precise and contextualized use of the Global North and South terms, aims to contribute to a broader conversation about fostering a psychology that is truly global, and inclusive, valuing the contributions of all regions and cultures.

In addressing the economies of knowledge production in CCP, we recognize the deeply embedded asymmetries that characterize the field of psychology. While it is crucial to highlight the imbalance in training, access, and influence that favors scholars from the Global North, it is equally important to acknowledge the complexity of these disparities. The underrepresentation of scholars from the Global South in high-status institutions and their relative absence in the setting of research agendas may stem from multifaceted factors, including but not limited to systemic resource imbalances, educational opportunities, and differing interests in psychological science paradigms. These challenges are not the result of intentional actions but often arise from longstanding structural inequalities that influence academic trajectories. Acknowledging these realities does not diminish the urgency of addressing these asymmetries but rather enriches our understanding of their roots and manifestations, guiding more nuanced and effective solutions to foster a more equitable and diverse scientific community.

The field of CCP needs a paradigm shift toward more equitable collaborations and knowledge co-production between researchers from different cultural and geographical backgrounds. Moreover, publication disparities significantly impact researchers from the Global South, affecting their representation and the dissemination of their work (Connell, 2007). Finally, a review paper on these topics has the potential to inform policy and practice, guiding institutions, funding bodies, and researchers in adopting more inclusive, equitable, and culturally sensitive approaches in CCP research.

### The legacy of colonialism and its implications for psychological scholarship

As a distinct discipline, cross-cultural psychology emerged in the mid-20th century, although its roots can be traced back to earlier anthropological and psychological inquiries (Berry et al., 2011). One of the early proponents, Gustave Le Bon, in his 1890s works, explored how cultural factors could influence individual and collective behavior. However, it wasn’t until the 1960s and 1970s that cross-cultural psychology began to establish itself firmly as an academic field, emphasizing empirical research methodologies that sought to compare and contrast psychological phenomena across cultures (Segall et al., 1999).

The growth of the discipline was accelerated by the global movements and migration patterns after World War II. These global shifts led to increased interactions among people from diverse cultural backgrounds, underscoring the need for understanding human behavior in a cross-cultural context. This period saw the emergence of significant cross-cultural studies, focusing on areas like cognition, emotion, and development, often revealing both universal and culture-specific aspects of human behavior (Triandis, 2007).

The legacy of colonialism casts a long shadow over various academic disciplines, including cross-cultural psychology. Historically, many Western scientists and scholars, consciously or unconsciously, approached non-Western cultures with a sense of superiority, perpetuating stereotypes and often misrepresenting or misunderstanding the cultures they studied (Said, 1979). These Eurocentric views framed non-Western societies as “primitive” or “underdeveloped,” resulting in biased interpretations of data and findings (Teo, 2010).

Furthermore, the very methodologies employed in early cross-cultural research were deeply rooted in Western paradigms. This often led to the inappropriate application of Western psychological instruments and scales in non-Western settings, without considering the cultural validity or relevance of these tools (Smith and Bond, 1993). Such practices did not just risk inaccurate findings but also contributed to reinforcing the dominance of Western perspectives in understanding human behavior globally.

Another critical implication of the colonial legacy in cross-cultural studies was the issue of intellectual extractivism. Western researchers, for decades, collected data from non-Western cultures, gaining academic accolades without necessarily benefiting or crediting the communities they studied. This dynamic not only marginalized scholars from the Global South but also deprived the broader academic community of rich, indigenous insights and understandings (Smith, 2012).

Intellectual extractivism can be described as the act of extracting knowledge, data, or intellectual resources from one culture or community, primarily without adequate recognition, compensation, or benefit to the source. This dynamic, echoing colonial resource extraction, shifts in the realm of knowledge where, instead of tangible resources like minerals, intellectual and cultural knowledge becomes the commodity. Especially in psychology, Western researchers may “mine” data from non-Western cultures and achieve academic milestones without significant involvement, acknowledgment, or compensation of the studied community or local scholars (Quijano, 2000; Smith, 2012).

Intellectual extractivism in psychology is reminiscent of colonial-era resource extraction. It not only involves “taking” knowledge but often imposes foreign interpretations, altering the essence of indigenous wisdom. Just as resource extraction disrupted local structures, extracting cultural knowledge without a two-way dialog risk fragmenting indigenous knowledge systems and perpetuating stereotypes (Césaire, 1955; Maldonado-Torres, 2007). The refined knowledge is often repackaged and “sold” back to its origins in the form of Western-validated theories, even if they might not be wholly relevant (Escobar, 1995).

More recently, Raval et al. (2023) explore the unique challenges faced by researchers conducting psychological research in Majority World communities, highlighting the historical neglect and bias against such research within the field of psychological science. Through an embedded mixed-methods design surveying 232 researchers, the study uncovers challenges related to inherent biases against Majority World research, amplified difficulties experienced by all researchers when working with Majority World populations, and specific obstacles for researchers affiliated with Majority World institutions. To promote a diverse and globally applicable psychological science, the authors recommend that journal editorial teams and funding agencies acknowledge and address these biases, recruit and train editorial members and reviewers from both Majority and Minority Worlds with sensitivity to Majority World research and provide additional resources to researchers from Majority World institutions. This study sheds light on the critical need for inclusivity and equity in psychological research practices and publications.

### Challenges of representation and interpretation

Cross-cultural psychology, while championing the understanding of diverse human behaviors, can inadvertently walk on the path of cultural misunderstandings. Such misinterpretations might arise when researchers, perhaps unintentionally, place their cultural norms or biases onto another culture.

#### The risks of cultural misunderstanding

In the field of psychology, the translation of assessment tools and questionnaires is a critical aspect of cross-cultural research. However, this process is fraught with challenges, as highlighted in the seminal works of Brislin (1986) and van de Vijver and Tanzer (2004). When psychological tools are translated from one language to another, there is a risk of semantic and conceptual discrepancies, which can significantly impact the validity and reliability of these instruments. Brislin (1986) underscored the complexities involved in the translation process. He noted that direct translation often fails to capture the nuanced meanings of certain terms and phrases inherent in the original language. These subtleties can be crucial in psychological assessments, where the precision of language is paramount. For instance, a word in one language might have multiple meanings or connotations in another, leading to varied interpretations among respondents. This variance can result in data that are not truly comparable across cultures or linguistic groups. Further expanding on these challenges, Van de Vijver and Tanzer (2004) discussed the implications of such translation issues on the psychometric properties of the tools. They argued that discrepancies in translation can lead to differential item functioning (DIF), where items on a test or questionnaire may function differently across different language versions. This differential functioning can compromise the construct validity of the measure, as it may no longer assess the same underlying concept across different groups. Moreover, these inconsistencies can also affect the tool’s reliability, as the variability introduced by translation issues can inflate error variance.

#### Ethnocentrism

In the field of psychology, and particularly in cross-cultural research, the concept of ethnocentrism plays a critical role in understanding how individuals perceive and evaluate cultures different from their own. Ethnocentrism, as described in psychological literature, refers to the tendency to view one’s own culture as the center of everything and to evaluate other cultures based on one’s own cultural norms and values (Sumner, 1906). This perspective can significantly distort the understanding of other cultures, leading to biased interpretations and conclusions in cross-cultural research. The concept of ethnocentrism was first thoroughly examined by Sumner (1906), who defined it as the viewpoint that one’s own group is the center of everything, and all others are scaled and rated with reference to it. He noted that this viewpoint leads to a sense of group superiority and a denigration of other cultures. In contemporary research, the implications of ethnocentrism are particularly significant in psychology. Berry (1969) emphasized how ethnocentrism can influence psychological research, especially when researchers fail to recognize their own cultural biases. This failure can lead to the development of theories and practices that are only relevant or valid within the researcher’s own cultural context, thereby limiting their universality.

Ethnocentrism can manifest in various aspects of psychological research, from the formulation of research questions to the interpretation of data. Nisbett (2003), in his work on cognitive differences between Eastern and Western cultures, highlighted how researchers often inadvertently apply their cultural norms and cognitive styles when designing studies and interpreting results. Moreover, ethnocentrism can lead to the misapplication of psychological constructs across cultures. Triandis (1994) pointed out that many psychological constructs are culture-bound and may not have the same relevance or meaning in different cultural contexts. By applying these constructs universally, researchers may end up misrepresenting the psychological phenomena they are studying.

#### The perpetuation and reinforcement of stereotypes

Stereotypes, broadly defined, are oversimplified generalizations about a group of people. While they may contain elements of truth, stereotypes often ignore the complexity and diversity within groups. Research has the potential to inadvertently feed into these stereotypes, especially when findings are either misconstrued or decontextualized. This issue is particularly pronounced in cross-cultural psychology, where studies focusing on differences between cultural or ethnic groups can inadvertently reinforce simplistic or negative perceptions of those groups (Sue, 1999). One seminal paper that discusses the risks associated with decontextualized research is by Sue (1999), who highlighted the dangers of drawing broad conclusions from data that do not consider the full cultural or situational context. Sue pointed out that such research can reinforce existing stereotypes and contribute to biases in both academic and public spheres.

Another perspective is offered by Oyserman and Lee (2008), who examined how the interpretation of research findings can be influenced by the prevailing stereotypes. They argued that researchers, often unconsciously, might frame their findings in ways that align with existing stereotypes, thus reinforcing them. Moreover, the impact of stereotypes in psychological research is not just limited to academic discourse. Media representations of research findings play a crucial role in how the general public perceives different groups. Oliver and Fonash (2002) demonstrated how media reports of psychological studies often simplify or sensationalize findings, leading to the reinforcement of stereotypes among the general public.

The reinforcement of stereotypes, especially in the context of cross-cultural psychology, can be critically analyzed through the lens of construal level theory. This theory suggests that the psychological distance between the self and others influences how abstractly or concretely individuals construe information, which in turn affects their readiness to stereotype and the accuracy of those stereotypes (Trope and Liberman, 2010). Research indicates that stereotypes are more readily applied and less accurate when they concern outgroups or those perceived as socially or geographically distant, a phenomenon that is exacerbated when such groups are less powerful or marginalized (Hansen et al., 2014; Kim et al., 2020).

This asymmetry becomes particularly problematic when psychologists from the Global North, representing an “ingroup” with more power and resources, make attributions about peoples from the Global South, the “outgroup,” without sufficient consideration for intra-cultural diversity. Such attributions risk oversimplifying the rich tapestry of human experience and perpetuating stereotypes that do not reflect the complex realities of individuals’ lives in these regions (Reitz and Banerjee, 2007; Reitz et al., 2009). To mitigate this issue, it is essential to apply a more nuanced understanding of construal level theory to psychological research, emphasizing the importance of recognizing and valuing intra-cultural diversity and challenging researchers to question their assumptions and biases when studying cultures other than their own (Brady et al., 2017).

#### Intra-cultural diversity

In the discipline of psychology, especially within cross-cultural studies, acknowledging the substantial variability within cultural groups is crucial. This intra-cultural diversity often surpasses the inter-cultural differences that are typically the focus of many studies. Ignoring this variability can lead to the fallacy of the “single story” narrative, where a culture is erroneously represented as monolithic and uniform, thereby oversimplifying and misrepresenting the true nature of the group. Moreover, overlooking intra-group variability can lead to significant issues in understanding and addressing mental health across different cultural contexts.

Sue and Sue (2016) highlight how diversity within cultural groups includes variations in socioeconomic status, religion, and individual experiences, all of which can influence mental health and its treatment. By ignoring these factors, psychologists risk applying generalized interventions that may not be effective for all individuals within a cultural group. The implications of the single-story fallacy extend beyond academic research to educational practices and policymaking. Banks (2016) emphasizes the importance of incorporating a multifaceted understanding of cultures in educational curricula to avoid perpetuating stereotypes and to foster a more accurate and inclusive understanding of cultural diversity among students.

The indigenous psychology movement can also facilitate this critical shift toward recognizing psychological practices and understandings that are sensitive to intracultural diversity and native to different cultures, outside of the Western paradigm. This movement underscores the significance of cultural context in the study of psychological processes and posits that psychological theories and practices cannot be universally applied without consideration of local cultural nuances (Kim et al., 2006). Indigenous psychology advocates for the development of psychological models that are rooted in the specificities of each culture’s historical, social, and cultural background, challenging the field to broaden its epistemological foundations beyond Western-centric models (Allwood and Berry, 2006).

By integrating indigenous psychology perspectives, researchers can gain a deeper understanding of the diversity of human psychology, acknowledging that what constitutes as normative behavior or thought in one culture may not necessarily hold in another. This approach not only enriches the field of psychology with a multiplicity of perspectives but also ensures that psychological research and practice are more inclusive and representative of global diversity (Adair, 2006; Adair and Huynh, 2012; Ali et al., 2012). The movement calls for a collaborative approach to knowledge production, where scholars from the Global North and South work together to co-create psychological knowledge that respects and incorporates indigenous epistemologies and methodologies (Enriquez, 1992). Medin and Bang (2014) and Cole and Vossoughi (2015) both emphasize the significance of incorporating cultural diversity into psychological and anthropological research to enhance understanding and effectiveness. Cole and Vossoughi highlight the necessity for a methodologically sophisticated approach that appreciates the role of culture in human development, drawing on studies contrasting Native American and European American orientations toward nature. Similarly, Medin and Bang argue that the cultural values and orientations of scientists influence their research, advocating for the inclusion of Native American perspectives to improve science and science education. They demonstrate that embracing diverse cultural perspectives, particularly those from historically marginalized communities, not only enriches scientific inquiry but also makes science education more inclusive and effective. Together, these works underscore the importance of cultural diversity in the scientific community, proposing that a more comprehensive and nuanced approach to science can be achieved through the integration of varied cultural insights (Medin and Bang, 2014; Cole and Vossoughi, 2015).

In acknowledging intra-cultural diversity within psychological studies, it’s essential to consider the complex dynamics of individual and collective experiences within cultural contexts. The experiment conducted by Anjum et al. (2020) in Pakistan, investigating the impact of United Nations endorsements on women’s rights, highlights the importance of international recognition in influencing domestic attitudes toward human rights reforms. This underscores the variability of responses within a single cultural group, shaped by perceptions of legitimacy and authority. Similarly, Anjum et al. (2019) in a cross-cultural exploration of honor in Germany, Pakistan, and South Korea reveal the nuanced ways in which cultural values and group dynamics influence individuals’ adherence to societal norms, further complicating the notion of a monolithic cultural identity within these cultures. These studies advocate for a more nuanced understanding of intra-cultural diversity, emphasizing the importance of considering a wide range of individual experiences, societal norms, and cultural values in psychological research and practice.

#### Cultural dynamism

In psychological and sociological research, the dynamic nature of cultures is a fundamental concept that must be recognized. Cultures are not static entities; they evolve and change over time. However, there is a risk in research methodologies that fail to take into account this fluidity. By not acknowledging the dynamic nature of cultures, research might inadvertently present a culture as monolithic and unchanging, effectively freezing it in a specific time and context. This oversight can lead to a skewed understanding of the culture and its members. Adams and Markus (2004) discussed this issue, emphasizing the importance of considering cultural changes over time in psychological research. They argued that when researchers overlook the dynamic aspects of culture, their work can perpetuate outdated stereotypes and misconceptions. This static portrayal of culture fails to capture the ongoing processes of cultural adaptation and change, resulting in a distorted view of the cultural group in question.

The concept of cultural dynamism is particularly relevant in the context of globalization and migration. Arnett (2002) highlighted how increased global interconnections have led to significant cultural changes, particularly in younger generations. Cultures today are influenced by a myriad of factors, including technology, media, and interactions with other cultures, all of which contribute to their evolving nature. Ignoring these influences can result in a misunderstanding of contemporary cultural practices and values. Furthermore, the failure to recognize cultural dynamism can have implications for cross-cultural comparisons in psychological research. Henrich et al. (2010a) cautioned against assuming cultural homogeneity when conducting cross-cultural studies. They noted that cultural groups often exhibit significant internal diversity and are subject to ongoing changes, which must be considered when comparing different cultures.

Moreover, the work by Khalid and Anjum (2019) on dyslexia in Pakistan, Furrukh and Anjum (2020) on autism spectrum disorder coping strategies, and Anjum (2020) on women’s activism within the frameworks of religious nationalism and feminist ideology, is important to understand the nuanced challenges and coping mechanisms within specific cultural contexts is crucial. These studies underscore the imperative for culturally tailored interventions and illustrate the rich tapestry of intra-cultural diversity that defies simplistic categorizations. Emphasizing cultural dynamism, this body of work highlights the importance of acknowledging the fluid and evolving nature of cultural identities, influenced by globalization, technological advancements, and intercultural exchanges. It advocates for a research paradigm that is adaptable and nuanced, capable of capturing the complex realities of cultures in flux. This approach not only enriches the psychological discourse but also ensures that interventions and theoretical models are reflective of the diverse and dynamic nature of human societies, urging a move away from static representations toward a more holistic understanding of cultural dynamism.

### Addressing regional specificity in cross-cultural psychology

The critique that cross-cultural psychology has predominantly focused on East–West comparisons, primarily between the United States and East Asian countries, highlights a significant limitation in the field’s approach to understanding global psychological diversity. This emphasis has inadvertently marginalized other regions, notably those within what is broadly categorized as the Global South, which encompasses a rich tapestry of cultures, societies, and historical backgrounds. Recent scholarship emphasizes the necessity of broadening the scope of cross-cultural research to include a more diverse range of cultures and regions, particularly those in Latin America and beyond, which have been underrepresented in psychological research.

Research by de Oliveira and Nisbett (2017) and Krys et al. (2022) underscores the unique cognitive styles and notions of self within Latin American societies, challenging the binary classification of collectivist versus individualist cultures. These studies reveal that Latin American cultures, while exhibiting collectivist social norms, simultaneously foster a sense of independence and self-expression. Furthermore, Krys et al. (2024) argue for the necessity of moving beyond the WEIRD–Confucian comparisons to include a wider array of cultural contexts in psychological research, advocating for a global representation that accurately reflects the world’s cultural diversity.

Kitayama and Salvador (2023) further advocate for a cultural psychology that transcends the East–West paradigm, emphasizing the importance of integrating diverse cultural perspectives to achieve a truly global understanding of human psychology. Their work, along with the contributions of others in the field, calls for an expansion of the cultural and regional scope of psychological research. This includes not only acknowledging but actively seeking to understand and represent the psychological dynamics of regions that have been historically overlooked or marginalized within the academic discourse.

These recent contributions to the field of cross-cultural psychology underscore the imperative for researchers to broaden their investigative lens to include regions beyond the traditional East–West comparisons. By doing so, the field can move toward a more inclusive, equitable, and comprehensive understanding of the complex tapestry of human culture and psychology. Incorporating these diverse perspectives not only enriches the discipline but also ensures that psychological science is reflective of and relevant to the global population it seeks to understand.

### Expanding epistemological diversity in cross-cultural psychology

The field of cross-cultural psychology is at a pivotal moment, with its theoretical underpinnings deeply rooted in Western perspectives, leading to the marginalization of the diverse and rich epistemologies from scholars in developing countries. This predominance narrows the scope of psychological inquiry and limits the understanding of human behavior across varied cultural contexts. Acknowledging this disparity is crucial for broadening the theoretical base of cross-cultural psychology, essential for fostering a more inclusive and globally representative science (Adams et al., 2015).

Language barriers and publishing criteria that favor Western methodologies pose significant challenges to incorporating theories from the Global South into mainstream psychology journals. This cycle keeps theories from developing countries on the periphery of psychological discourse, despite their potential to offer invaluable insights (Henrich et al., 2010b). Yet, there is a wealth of theoretical contributions from these scholars that can significantly enrich cross-cultural psychology. For instance, the Ubuntu philosophy highlights interconnectedness and community, offering a framework for understanding behavior that contrasts with Western individualism (Mbiti, 1990). Indigenous psychologies also provide unique perspectives on identity and mental health, advocating for psychological concepts to be understood through local cultural traditions and values (Kim et al., 2006).

Integrating these diverse theoretical frameworks into cross-cultural research diversifies perspectives and challenges the universality of psychological constructs. This incorporation is not merely about academic inclusivity; it’s crucial for developing a psychology reflective of human experience. Diverse epistemological backgrounds can lead to more culturally sensitive research methodologies and nuanced data interpretations, such as methodologies that incorporate storytelling and narrative analysis, offering deeper cultural insights (Gregg, 2007).

To advance, cross-cultural psychology must encourage the publication of work from outside Western contexts, support scholars from the Global South, and promote collaborative research that bridges cultural divides. This evolution is vital for building a knowledge base that truly represents how culture shapes psychological processes, making cross-cultural psychology a discipline that values the diversity of psychological science (Allwood and Berry, 2006).

#### Balancing epistemological diversity with scientific standards

Recent scholarship in cross-cultural psychology underscores the importance of integrating diverse epistemologies while maintaining rigorous scientific standards. The discourse around what constitutes as (good) science is increasingly acknowledging the value of methodological pluralism and epistemological diversity, particularly to enhance the field’s comprehensiveness and relevance across different cultural contexts (Henrich et al., 2010b). This emerging consensus suggests that embracing a variety of research methodologies and theoretical perspectives can contribute to a more robust and nuanced understanding of psychological phenomena. However, the challenge lies in balancing these diverse approaches with the necessity of maintaining scientific rigor. This task that demands careful consideration of replication, theoretical coherence, and methodological integrity (Nosek et al., 2018).

The call for more inclusive epistemological frameworks extends beyond mere critique; it emphasizes the development of collaborative research models that empower scholars from the Global South. Initiatives like participatory action research (PAR) highlight the potential for equitable research partnerships that respect local knowledge and expertise while pursuing scientifically rigorous outcomes (Omodan and Dastile, 2023). Such models prioritize reciprocal learning and shared decision-making, thereby challenging paternalistic dynamics that have historically characterized some cross-cultural research endeavors.

Addressing the practical challenges associated with implementing these inclusive and collaborative approaches requires innovative solutions. For instance, open access models, while aimed at democratizing access to scientific knowledge, often inadvertently impose financial burdens on researchers from under-resourced regions. Recent proposals suggest a tiered fee structure for publication charges, which would consider the economic disparities between regions, ensuring that scholars from the Global South can participate in the global scholarly discourse without undue financial hardship (Jhangiani and Biswas-Diener, 2017).

It is also pertinent to acknowledge some of the existing models that embody principles of financial inclusivity within the academic community. Notably, the Asian Association of Social Psychology (2024) and International Association for Cross-Cultural Psychology (2024) have pioneered tiered fee structures for membership dues, reflecting a commitment to inclusivity and diversity. The IACCP’s fee structure is thoughtfully adjusted based on the economic level of a member’s country, aiming to ensure that scholars from lower-income regions can participate fully in the association’s activities without facing prohibitive costs. Similarly, the AASP implements a tiered membership policy, which serves as a testament to the association’s dedication to fostering a diverse and inclusive academic environment that accommodates scholars from various economic backgrounds (Asian Association of Social Psychology, 2024). These initiatives exemplify practical steps toward mitigating the disparities in academic participation and publishing, offering valuable precedents for the implementation of a tiered fee structure for publication charges. By adopting such models, scholarly publishers can contribute significantly to leveling the playing field, thereby advancing the global and cross-cultural exchange of knowledge and enhancing the diversity of perspectives within the field of psychology.

Moreover, the development of global research consortia and networks can facilitate more balanced contributions from scholars across different regions, promoting a more equitable distribution of voice, power, and representation within the academic community. These networks can serve as platforms for mentoring, resource sharing, and collaborative project development, effectively addressing some of the asymmetries in training, access, and influence that currently exist (Muthukrishna and Henrich, 2019; Muthukrishna et al., 2021; Krys et al., 2024).

Overall, while the integration of diverse epistemologies and collaborative research models presents complex challenges, it also offers a pathway toward a more inclusive, equitable, and scientifically rigorous cross-cultural psychology. By actively engaging with these issues and seeking out innovative solutions, the field can move closer to realizing its potential as a truly global discipline that values and benefits from the richness of human diversity.

## The economics of knowledge production in CC psychology

CC Psychology, as a discipline that seeks to understand human behavior in diverse cultural contexts, finds itself at a pivotal junction of knowledge dissemination and creation. However, beneath its noble aspirations, psychology is influenced by complex economic, historical, and geopolitical factors that often remain unacknowledged. These factors when explored show significant issues, such as the pronounced influence of the Global North in dictating research agendas and funding, alongside the challenges that scholars from the Global South encounter in accessing vital scholarly resources. Such exploration sheds light on the underappreciation of contributions from researchers in the Global South, who bring indispensable local contexts and insights to the table (Patel, 2014).

The dominance of the Global North in psychological research is a well-established concern, highlighting a skewed focus toward WEIRD populations, primarily from the United States and Europe. This bias is not limited to research subjects but extends to the control over research agendas and funding allocations, predominantly managed by institutions within these regions (Arnett, 2008; Krys et al., 2022, 2024). This imbalance fosters a narrow representation of human behavior, often generalizing findings from Western populations to a global scale, thus overlooking the rich diversity of human experience.

In parallel, researchers from the Global South face substantial barriers in engaging with and contributing to the global scholarly discourse. Liu et al. (2023) reveal that non-White scientists encounter systemic inequalities that hinder their participation in academia, from disparities in editorial board representation to prolonged manuscript review periods, and lower citation rates for their published work. Despite facing these obstacles, the contributions of scholars from the Global South remain invaluable. Their unique perspectives and insights are critical for developing a truly global understanding of psychology, emphasizing the need for culturally relevant psychological interventions (Patel, 2014).

In the domain of global research, the allocation and focus of funding by prominent bodies in the Global North significantly shape the research agendas, often leading to a divergence from the pressing issues and needs of communities in the Global South. This misalignment, as highlighted by Tikly (2004) and Connell (2007), stems from the disproportionate influence these funding organizations wield, prioritizing research themes and methodologies that reflect their own interests and perspectives. This scenario not only impacts the relevance and applicability of research outputs but also underscores a systemic issue where the priorities of the Global North overshadow those critical to the development and well-being of the Global South. The dominance of research funding from regions such as the United States and Western Europe, as documented by King (2004), exacerbates this issue by concentrating resources in a manner that often neglects local health issues, environmental challenges, and the valorization of indigenous knowledge systems that are paramount to the Global South.

This prevailing focus of research funding and agendas has tangible repercussions on the ground. Bradley (2007) and Mignolo (2012) point out that the misalignment between funded research priorities and the actual needs of communities in the Global South leads to an oversight of critical health issues unique to these regions, alongside a broader marginalization of non-Western epistemologies and methodologies. This epistemic dominance not only neglects the socio-economic conditions and health concerns prevalent in the Global South but also contributes to a homogenization of knowledge production. Such a trend underlines the urgent need for a recalibration of research priorities and funding practices to ensure they are inclusive, relevant, and responsive to the diverse realities and challenges faced by communities globally, thereby fostering a more equitable and representative body of global research.

### Dependency and autonomy

The dependence on resources from the North can create a cycle where scholars from the Global South might tailor their research to fit these interests, potentially sacrificing local relevance or autonomy (Quijano, 2000; Alatas, 2003; Connell, 2007; Mignolo, 2012). In the field of academic research, particularly within cross-cultural psychology, the dependence on resources from the Global North can create a cyclical pattern that significantly influences the nature and direction of research conducted by scholars in the Global South. This dependence often leads to a situation where these scholars may feel compelled to align their research interests with those favored by Northern funding bodies. Such a dynamic poses a substantial risk of compromising the local relevance and autonomy of their research.

The impact of this dependence is multifaceted. One critical aspect is the potential loss of local relevance in research. As scholars in the Global South vie for funding from predominantly Northern institutions, there is a tendency to prioritize topics and methodologies that are more likely to receive support, even if they do not align with local needs or contexts. This issue is highlighted by Smith (2012) in her discussion on indigenous methodologies. She points out that research in indigenous communities often fails to reflect the priorities and values of these communities, largely due to the influence of external funding sources and agendas.

Another aspect is the erosion of academic autonomy. Dependency on Northern resources can lead scholars in the Global South to adopt research frameworks and methodologies that are not inherently suited to their cultural and social contexts. This concern is echoed in the work of Santos (2014), who discusses the necessity of developing a more pluralistic epistemology that respects and integrates diverse ways of knowing, particularly those originating from the Global South.

The cycle of dependency also has broader implications for the development of a truly global psychology. As Teo (2010) argues, psychology’s history and current practices are predominantly shaped by Western perspectives, which can limit its relevance and applicability in diverse cultural settings. This Western-centric approach risks marginalizing non-Western perspectives and knowledge systems, thereby limiting the field’s ability to address the psychological needs and realities of diverse populations.

### Disparity in access to published research outputs

The issue of accessibility to scholarly publications, particularly accentuated by subscription-based models, presents a significant barrier to the dissemination and utilization of scientific knowledge, disproportionately impacting researchers in the Global South (Chan and Costa, 2005; Larivière et al., 2015). These models, largely managed by major publishers situated in the Global North, necessitate substantial fees for access, creating an unequal landscape where access to the latest scientific advancements is contingent upon one’s institutional or personal financial capacity. Larivière et al. (2015) highlight the centralization of scientific publications under a few major publishers, which exacerbates this access disparity by restricting the availability of scientific literature to those able to afford the exorbitant subscription fees. This system inherently disadvantages researchers from less affluent backgrounds, particularly those in the Global South, limiting their participation in and contribution to the global scientific dialog.

The repercussions of this limited accessibility are profound, as elucidated by Chan and Costa (2005), who argue that the resultant knowledge gap hinders the progression of scientific inquiry in less economically developed regions. This gap perpetuates a cycle of academic dependency and inequality, stifling the potential for a truly global scientific community that leverages diverse insights and contributions. The restriction of access to scholarly publications not only undermines academic equity but also impedes the advancement of science and the formulation of solutions to global challenges. Willinsky (2006) further asserts that the limited dissemination of research findings hampers the global research community’s capacity to build upon existing knowledge, thus slowing scientific innovation and discovery.

Conversely, open access (OA) publishing emerges as a potential antidote to these challenges, aiming to democratize access to scholarly knowledge by eliminating subscription barriers. However, this model introduces new challenges, notably financial barriers for researchers, especially those from the Global South, in the form of article processing charges (APCs) (Tennant et al., 2016; Morrison, 2017; Piwowar et al., 2018). While OA aims to enhance the visibility and impact of research by making it freely available, the associated costs of APCs can be prohibitive for researchers from underfunded institutions or lower-income countries, as noted by Björk and Solomon (2012). This financial hurdle poses a significant obstacle to the equitable dissemination of research findings, threatening to reinforce the disparities OA seeks to overcome.

Addressing the financial and accessibility disparities inherent in the current publishing models requires a multifaceted approach. Solutions such as differentiated pricing models for APCs, increased funding support for researchers in the Global South, and the development of sustainable OA models that equitably distribute publication costs are imperative (Chan, 2004; Vincent-Lamarre et al., 2016). Implementing these measures can mitigate the financial barriers faced by researchers in the Global South, fostering a more inclusive academic landscape. Such initiatives are crucial for realizing the potential of open access to democratize academic research, ensuring that the global scientific community is reflective of and accessible to scholars from diverse economic and geographical backgrounds.

### Economic inequality of scholars and researchers from the global south

The discourse on the economic exploitation of scholars and researchers from the Global South elucidates a complex interplay of unpaid labor and skewed authorship attribution, underpinning a broader narrative of inequity within academic research collaborations. The critical contributions of local scholars, particularly in ensuring the cultural sensitivity and relevance of cross-cultural studies, are indispensable. These scholars offer invaluable insights through data collection and cultural understanding, which form the backbone of valid and relevant research outcomes. Despite their pivotal role, the prevailing academic model often fails to provide equitable compensation or recognition, particularly in collaborations between the Global North and South, thus undermining the integrity and quality of research (Smith, 2012; Czerniewicz, 2013).

The under compensation of local scholars is a pronounced issue, especially in lower-income countries. Birn et al. (2011) highlighted the challenges faced by these researchers in securing fair compensation and recognition for their contributions. The systemic undervaluation not only impacts their livelihoods but also detracts from the principle of equitable collaboration in global research endeavors. This disparity extends beyond financial remuneration, affecting academic recognition and the equitable distribution of authorship in research publications. Such hierarchical structures in academic publishing exacerbate the marginalization of researchers from the Global South, calling into question the fairness and integrity of collaborative research practices.

Authorship attribution further complicates the recognition and valuation of contributions from local researchers. The nuanced dynamics of authorship often overshadow the significant roles played by these scholars, particularly in collaborative projects that span geographical and cultural divides. Studies by Rossiter (1993), Heine et al. (2009), and Crane (2017) underscore the ethical considerations surrounding authorship, emphasizing the need for fair and equitable practices that reflect the academic currency and career advancement tied to these acknowledgments. The tendency to relegate local researchers to lesser roles or omit them from authorship not only fails to acknowledge their contributions but also perpetuates a colonial legacy within academic research.

The influence of editorial boards, predominantly based in the Global North, on the landscape of psychological research introduces a gatekeeping mechanism that biases the representation and valuation of research contributions. A study by Goyanes and Demeter (2020) revealed a significant correlation between the diversity of editorial boards and the diversity of research articles, indicating the profound impact of editorial practices on the inclusivity and diversity of academic discourse. However, the underrepresentation of the Global South on these boards limits the discipline’s ability to incorporate culturally specific phenomena and indigenous knowledge systems. Pieterse (2015) argues for the inclusion of diverse perspectives to address the psychological needs and realities of a global population comprehensively.

Addressing the economic exploitation and inequities in authorship practices requires a concerted effort to reform the structures and norms that govern academic collaborations and publications. Fair compensation, equitable collaboration, and the recognition of all researchers’ contributions, irrespective of their geographical location or institutional affiliation, are essential for fostering a more inclusive and respectful academic environment. By ensuring a more balanced representation on editorial boards and advocating for equitable authorship practices, psychology can advance as a discipline that truly reflects the diverse human experiences and cultures it seeks to understand. Smith (2021) emphasizes the importance of embracing and integrating diverse perspectives and knowledge systems, underlining the potential of psychology to evolve into a global discipline that values contributions from all regions equally.

In addressing the critical issue of unpaid labor and financial compensation within cross-cultural psychology, it is essential to navigate the complexities and ambiguities inherent in establishing equitable practices across diverse economic and cultural landscapes. The challenge of determining appropriate compensation extends beyond the mere acknowledgment of asymmetric financial remuneration, delving into deeper questions about the interplay between economic disparities, cost of living differences, and varied cultural values around monetary versus non-monetary rewards. The global diversity in GDPs and living standards raises important considerations about whether uniform compensation is feasible or desirable, and how such financial incentives might influence scientific motivations and the authenticity of research participation. Moreover, the ethical implications of compensating research contributors in lower-income, non-market economies necessitate a careful examination of existing literature and practices to navigate these dilemmas thoughtfully. While advocating for more equitable compensation structures, it is imperative to engage with the broader academic and ethical discourse on this issue, recognizing that solutions must be tailored to address the specific contexts and needs of scholars and participants from the Global South. This approach not only underscores our commitment to fairness but also acknowledges the intricate factors that shape compensation practices in cross-cultural research endeavors.

Regarding asymmetries in authorship and attribution between scholars from the Global North and South, we recognize the importance of examining the systemic and institutional norms that may inadvertently favor contributions traditionally recognized within Western academic infrastructures. These norms, often well-intentioned and designed to clarify the roles and contributions of co-authors, can inadvertently marginalize invaluable contributions from informants, translators, and local collaborators who play critical roles in cross-cultural research. These contributors provide essential insights and content that are fundamental to the research’s success but may not align with the conventional definitions of scientific production. We acknowledge that the issue of equitable attribution extends beyond the intentions of individual researchers and is embedded within broader institutional structures that have historically prioritized certain types of contributions. By addressing these structural biases, we aim to highlight the need for a more inclusive understanding of what constitutes meaningful contribution in cross-cultural psychology, one that values the diverse roles and insights that collaborators from the Global South bring to the research process. This adjustment not only seeks to rectify inequities but also enriches the scientific discourse by ensuring a broader range of perspectives and experiences are recognized and valued.

### Strategies for equitable collaboration

In addressing the evolving dynamics within cross-cultural psychology, the imperative to incorporate diverse voices and perspectives necessitates a strategic approach toward equitable collaboration between scholars from the Global North and South. This synergy not only enriches the research process but also ensures that outcomes are reflective of a comprehensive understanding of varied cultural contexts. Strengthening local and regional scholarly networks emerges as a foundational strategy, enabling the consolidation of knowledge within the Global South and fostering connections that are vital for the visibility and capacity building of scholars in these regions (Teferra and Altbachl, 2004; Mama, 2007). Such networks, alongside regional conferences and workshops, provide essential platforms for sharing research, receiving feedback, and establishing professional networks that prioritize local research agendas and methodologies.

The valorization of local publication outlets is another critical aspect of fostering equitable collaboration. By prioritizing platforms that resonate with and are relevant to specific communities, scholars can ensure their research is both impactful and meaningful within their local context, while also bridging the gap to global scholarship (Chan and Costa, 2005). Furthermore, the development of clear collaboration agreements at the outset of research projects is essential for delineating roles, responsibilities, and ensuring all contributions are adequately recognized, thereby addressing potential power imbalances and fostering a mutual setting of research agendas and equitable resource distribution (Smith and Ward, 2000; Bradley, 2007).

Local mentorship and training within the Global South are indispensable for nurturing the next generation of scholars. These initiatives, by leveraging the expertise of seasoned researchers familiar with local contexts, provide guidance in navigating the academic landscape and enrich the research process with region-specific methodologies and ethical considerations (Manuh et al., 2007; Nyamnjoh, 2012). Such efforts are crucial for ensuring that research remains culturally pertinent and methodologically sound.

Transitioning to recommendations for collaborators from the Global North, it becomes clear that genuine collaboration is rooted in trust, mutual respect, and the recognition of all partners’ expertise. This foundation is crucial for establishing respectful partnerships that value the unique contributions of each collaborator (Lave and Wenger, 1991). Additionally, mutual capacity building, recognizing indigenous methodologies, rectifying economic imbalances, and ensuring fair representation in publication are paramount. These actions, highlighting the necessity of equitable resource distribution and valuing local epistemologies, are essential steps toward mitigating the disparities and power dynamics historically prevalent in cross-cultural psychology research collaborations (Tervalon and Murray-García, 1998; Bradley, 2007; Mama, 2007; Smith, 2012; Crane, 2017; Hall and Tandon, 2017).

By integrating these strategies and recommendations into a unified approach, the field of cross-cultural psychology can move toward a more inclusive, equitable, and collaborative research environment. This necessitates not only a recognition of the existing disparities but also a commitment to actionable steps that respect the diversity of knowledge, methodologies, and perspectives. Such an integrated framework ensures that cross-cultural psychology remains true to its ethos, embodying a discipline that values and benefits from the richness of global diversity.

#### Integrating global south perspectives in cross-cultural psychology

Cross-cultural psychology has greatly benefited from the incorporation of diverse voices, particularly those from the Global South, whose contributions have significantly enhanced the field’s methodological diversity and theoretical depth. Scholars from these regions have introduced innovative concepts and methodologies that challenge the dominant paradigms and offer a more nuanced understanding of psychological phenomena across different cultural contexts. The work of scholars like Misra and Gergen (1993) in India, for example, has been pivotal in introducing the concept of relational subjectivity, challenging the individualistic perspectives prevalent in Western psychology. This approach has opened new avenues for exploring self-concept and identity within collectivist societies, emphasizing the importance of relational contexts.

Furthermore, methodological innovations developed by researchers in the Global South, such as those documented by Adair et al. (2002), Adair and Huynh (2012), have advocated for culturally sensitive research designs that take into account local languages, values, and social structures. These methodologies have contributed to a methodological pluralism within the field, encouraging research designs that are more aligned with the cultural nuances of the populations being studied. Similarly, scholars like Ali et al. (2012), and Nsamenang (1992) have challenged the universality of Western developmental psychology models, providing critical insights into child-rearing practices in African societies and highlighting the communal and interdependent nature of child development in these contexts.

The efforts of Global South scholars to promote equity in academic publishing have also been crucial in addressing the visibility and accessibility disparities within academic discourse. Initiatives like the establishment of the African Journal of Psychology serve not only to elevate the research conducted in these regions but also to enrich the global academic dialog with diverse perspectives and findings. Moreover, the advocacy for inclusive epistemologies by scholars such as Mkhize (2004), who has integrated Ubuntu philosophy into psychological practice, underscores the necessity of expanding the field’s theoretical frameworks to encompass non-Western philosophies and understandings of the mind and behavior.

These contributions underscore the indispensable role of Global South perspectives in fostering a more inclusive, equitable, and comprehensive field of cross-cultural psychology (Altbach, 2007). Recognizing and integrating these perspectives not only enriches the discipline but also ensures that it remains relevant and responsive to the diverse global population it seeks to understand.

Based on this review and the lived experience of the researchers, here are some actionable strategies that can be incorporated in psychological research to make it more equitable and accessible for the scholars from the Global South:

- Create funding opportunities specifically designed for collaborations between scholars from the Global North and South, with a focus on projects that address issues relevant to the Global South. These grants should prioritize equal partnership in all research phases.
- Journals should adopt policies that actively promote the publication of research from the Global South. This could include fast-tracking submissions from Global South scholars, waiving publication fees, and establishing quotas for Global South research articles.
- Launch training and mentorship programs that pair early-career researchers from the Global South with experienced scholars from the Global North. These programs should focus on building research skills, writing for publication, and grant writing, tailored to the needs of Global South researchers.
- Support and fund platforms, journals, and conferences that highlight and value indigenous methodologies and epistemologies. Encourage the integration of these methodologies in mainstream psychological research.
- Establish programs that facilitate scholars from the Global North working in Global South institutions and vice versa. These should aim at mutual knowledge exchange, capacity building, and fostering long-term collaborations.
- Ensure that research projects in the Global South are led by local researchers, with scholars from the Global North in supporting roles. This shift in leadership dynamics acknowledges and leverages the expertise of local scholars.
- Develop and disseminate clear ethical guidelines for cross-cultural collaboration that address power imbalances, authorship, financial compensation, and the valorization of all contributions, ensuring these guidelines are universally adopted.
- Financially support open access publishing for scholars from the Global South to ensure their work is accessible. This could include establishing funds specifically for article processing charges.
- Organize special issues in established journals focused on research from the Global South and issues of decolonization in psychology. Additionally, create awards and recognitions for outstanding contributions to cross-cultural psychology from Global South researchers.
- Encourage universities and research institutions in the Global North to recognize and value collaborative work with the Global South in tenure and promotion processes. This involves reevaluating what is considered valuable academic output and contribution.
- Implementing these practical solutions requires a concerted effort from all stakeholders in the psychological research community, including researchers, academic institutions, funding bodies, and scholarly journals. By adopting these strategies, the field of cross-cultural psychology can make significant strides toward a more inclusive, equitable, and decolonized discipline.

## Conclusion

The invaluable role of cross-cultural psychology in decoding human behavior across diverse cultural matrices remains unquestioned. However, the field is marred by imbalances and exploitative tendencies that recall colonial-era dynamics. The intricate challenges faced by Global South scholars, from the pitfalls of intellectual extractivism to the overshadowing presence of Western research methodologies, have been critically examined in this paper. Additionally, the potent influences that shape research trajectories, notably the overarching frameworks of the Global North’s financial and institutional systems, pose significant challenges for representative research rooted in the Global South’s realities. Such constraints not only limit genuine cultural representations but also hinder the broader aspirations of the discipline: a comprehensive grasp of human nuances across diverse cultural spectrums.

Reflecting on the discipline’s future trajectory, it’s evident that a paradigm shift is imperative, one that actively rebalances historical and entrenched inequalities. This transformation is not a mere rectification of past misjudgments but a proactive endeavor to make the discipline progressive, encompassing, and genuinely reflective of the global cultures under its purview. Central to this evolution is the accentuation of mutual respect, comprehension, and genuine collaboration. Every culture, irrespective of its global standing or historical narrative, is a treasure trove of wisdom and insights. Recognizing and valuing these contributions is essential.

Collaborative engagements should transcend superficial inclusions, emphasizing genuine partnerships marked by equality and holistic integration. By fostering such authentic collaborations, cross-cultural psychology can unveil profound insights, unburdened by a singular, often Western-biased, viewpoint.

It is thus an urgent call to action for the academic world, spanning both the Global North and South, to collectively challenge and dismantle these dominating practices. This path toward inclusivity, representation, and equity in cross-cultural psychological research promises a discipline that is not just comprehensive but also universally resonant, amalgamating the diverse threads of human experience into a rich mosaic of global cultures and psychologies.

Last but not least, it is also important to recognize the limitations of the Global North–South dichotomy and its potential for oversimplification. This framework, while useful for highlighting broad patterns of inequality, cannot capture the full complexity of global diversity. Our use of this dichotomy is intended as a heuristic tool to broadly categorize and discuss global inequalities while acknowledging the diversity within these regions. Moving forward, this categorization is open to refining further analysis to better reflect the complexities of global dynamics in the cross-cultural context, ensuring that the use of this dichotomy discussion contributes constructively to the broader conversation about equity and justice in psychological science cross-cultural debates.

## Author contributions

GA: Conceptualization, Funding acquisition, Investigation, Project administration, Resources, Visualization, Writing – original draft, Writing – review & editing. MA: Conceptualization, Investigation, Resources, Writing – original draft, Writing – review & editing.
